# Itaya virus, a Novel *Orthobunyavirus* Associated with Human Febrile Illness, Peru

**DOI:** 10.3201/eid2105.141368

**Published:** 2015-05

**Authors:** Robert D. Hontz, Carolina Guevara, Eric S. Halsey, Jesus Silvas, Felix W. Santiago, Steven G. Widen, Thomas G. Wood, Wilma Casanova, Nikos Vasilakis, Douglas M. Watts, Tadeusz J. Kochel, Hideki Ebihara, Patricia V. Aguilar

**Affiliations:** United States Naval Medical Research Unit No. 6, Lima, Peru (R.D. Hontz, C. Guevara, E.S. Halsey, D.M. Watts, T.J. Kochel);; University of Texas Medical Branch, Galveston (J. Silvas, F.W. Santiago, S.G. Widen, T.G. Wood, N. Vasilakis, P.V. Aguilar);; Institute for Human Infections and Immunity, Galveston, Texas, USA (J. Silvas, F.W. Santiago, N. Vasilakis, P.V. Aguilar);; Direccion Regional de Salud Loreto, Iquitos, Peru (W. Casanova);; Center for Biodefense and Emerging Infectious Diseases, Galveston (N. Vasilakis, P.V. Aguilar);; National Institute of Allergy and Infectious Diseases, National Institutes of Health, Rocky Mountain Laboratories, Hamilton, Montana, USA (H. Ebihara)

**Keywords:** Itaya, viruses, *Orthobunyavirus*, bunyavirus, Caraparu virus, human pathogen, febrile surveillance, Amazon, Peru, arboviruses, vector-borne infections

## Abstract

Our genetic analyses of uncharacterized bunyaviruses isolated in Peru identified a possible reassortant virus containing small and large gene segment sequences closely related to the Caraparu virus and a medium gene segment sequence potentially derived from an unidentified group C orthobunyavirus. Neutralization tests confirmed serologic distinction among the newly identified virus and the prototype and Caraparu strains. This virus, named Itaya, was isolated in 1999 and 2006 from febrile patients in the cities of Iquitos and Yurimaguas in Peru. The geographic distance between the 2 cases suggests that the Itaya virus could be widely distributed throughout the Amazon basin in northeastern Peru. Identification of a new *Orthobunyavirus* species that causes febrile disease in humans reinforces the need to expand viral disease surveillance in tropical regions of South America.

## Abstract 2

Analysis of uncharacterized bunyavirus isolates identified a possible reassortant virus.

The *Orthobunyavirus* genus, part of the group of viruses known as arboviruses, comprises several human and zoonotic pathogens known to be transmitted by mosquitoes, culicoides midges, nest bugs, and ticks and is the largest of the 5 genera within the *Bunyaviridae* family. Orthobunyaviruses, like other members of the *Bunyaviridae* family, have a trisegmented (large [L], medium [M], and small [S] segments) negative-sense RNA genome. The L RNA segment encodes for the RNA-dependent RNA polymerase, the M segment encodes for the glycoproteins Gn and Gc, and the S segment encodes for the nucleocapsid protein. Many orthobunyaviruses also encode the nonstructural proteins NSm and NSs within the M and S segments, respectively; however, these proteins are not encoded in all orthobunyaviruses described (*1*,*2*). 

Because of the segmented nature of their genome, bunyaviruses, like other segmented genome viruses, can undergo genetic reassortment. In recent years, increasing numbers of reassortant bunyaviruses have been identified by using sequencing and phylogenetic analyses, and novel reassortant bunyaviruses with increased pathogenicity have been documented (*3*,*4*). Evidence that genetic reassortment appears to be the driving force in bunyavirus evolution (*5*) strongly supports the possibility that novel reassortant bunyaviruses will continue to be identified. Therefore, efforts to characterize existing and recently isolated bunyavirus strains are needed.

Some of the viruses within the genus *Orthobunyavirus*, including Oropouche, Iquitos, Guaroa, Jamestown Canyon, La Crosse, Cache Valley, Wyeomyia, and members of the group C viruses such as Caraparu and Murutucu, have been documented as causes of clinical disease in humans in the Americas (*6*–*10*). These orthobunyaviruses cause many symptoms, primarily febrile illness that has potential to be severely debilitating and that is sometimes accompanied by neurologic manifestations requiring intensive care (*10*). Human group C viruses infections, largely associated with mild febrile illness, are indistinguishable from dengue fever (*9*), and recent studies on the genetic characterization of reference strains have described their genetic relationship (*11*).

Since 1999, the US Naval Medical Research Unit No. 6 (NAMRU-6) in Lima, Peru, has collaborated with the Peruvian Ministry of Health to investigate the etiology of febrile illnesses in Peru and greater Latin America (*9*). As part of these activities, >54 orthobunyaviruses, including group C, Guaroa, Maguari, and Oropouche viruses, were isolated, and some have been genetically characterized in an effort to understand their relationships to other strains identified in South America (*8*,*11*,*12*). These efforts have already resulted in identification of Iquitos virus as a proposed reassortant bunyavirus in the Simbu serogroup that causes febrile illness in Peru (*8*). 

A recent study that examined some clinical isolates of group C viruses in South America isolated during 2003–2008 showed that the strain FSL2923, isolated from a febrile patient in Yurimaguas in 2006, had complete L and S RNA segments of Caraparu virus; however, the M segment was only 75.3% identical to that of Caraparu (*11*). No attempts were made to antigenically characterize the strain to confirm differences from the Caraparu virus. Here, we report the identification of this strain as a possible novel reassortant group C virus, which we named Itaya virus after the Itaya River that surrounds Iquitos, where this virus was isolated. We demonstrate that the Itaya virus causes clinical disease in humans similar to that of other group C viruses. We also describe the genetic relationship of Itaya virus to other group C serogroup viruses and the clinical manifestations among persons infected with the viruses. 

## Methods

### Viruses

The viral isolates used in this study are summarized in Table 1. The first strain of Itaya virus (IQT9646) was isolated in 1999 from samples from a 25-year-old man in Iquitos, Peru. The second strain (FSL2923) was isolated in 2006 from a 59-year-old febrile man in Yurimaguas, Peru (*11*). The origin of the Caraparu strain BeAn3994 was described by Causey et al. (*16*). The source and isolation of group C virus prototype strains have been described elsewhere (*9*,*17*,*18*).

**Table 1:** Viruses analyzed to determine their genetic relationship to Itaya virus, a novel *Orthobunyavirus*, Peru*

Strain	Country	Year isolated	Host species	Age, y/sex	Occupation	Virus	Reference
IQT9646	Peru	1999	Human	25/M	Worker	Itaya	This study
FSL2923	Peru	2006	Human	59/M	Teacher	Itaya	(*11*)
FPI2066	Peru	2011	Human	29/M	Farmer	Caraparu	This study
BeAn 3994	Brazil	1956	Sentinel monkey	NA	NA	Caraparu	(*13*,*14*)
BeAn 974	Brazil	1955	*Cebus apella*	NA	NA	Murutucu	This study
TRVL 51144	Trinidad	1963	*Culex (Melanoconium) portesi*	NA	NA	Restan	This study
TRVL 18462	Trinidad	1957	*Culex (Aedinus) accelerans*	NA	NA	Nepuyo	This study
77V-74814	Brazil	1977	Sentinel mouse	NA	NA	Broconha	This study
Fe3–71H2	United States	1963	*Culex* (Melanoconium)	NA	NA	Gumbo Limbo	This study
BeAn 848	Brazil	1955	*Cebus apella*	NA	NA	Apeu	(*15*)
BeAn15	Brazil	1954	*Cebus apella*	NA	NA	Marituba	(*11*)
BeAn17	Brazil	1954	*Cebus apella*	NA	NA	Oriboca	(*11*)
BT4075	Panama	1961	Human	36/M	Unknown	Madrid	(*11*)

*NA, not applicable.

### Study Sites

**Figure 1:**
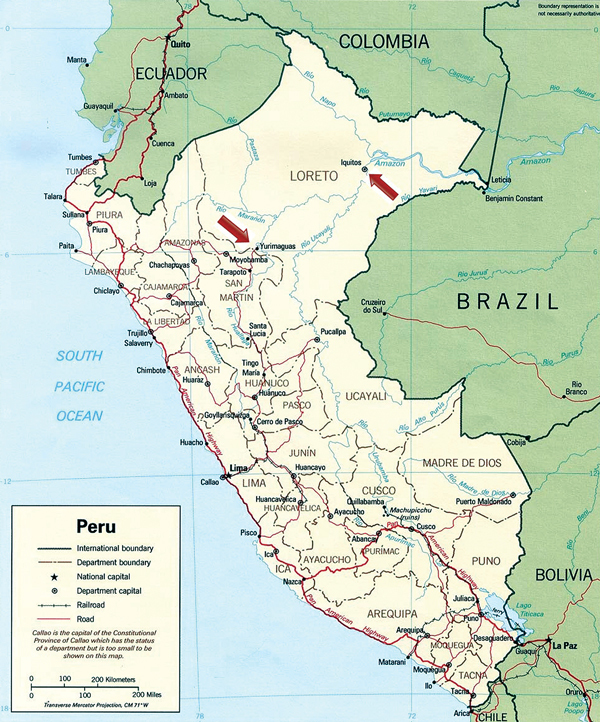
Geographic distribution of the confirmed Itaya virus human cases (arrows) identified as part of the febrile disease surveillance project in Loreto, Peru during 1999 and 2006

The locations of the study sites where IQT9646 and FSL2923 were isolated are depicted in Figure 1. Iquitos is a city of ≈380,000 inhabitants located 120 meters above sea level in the Amazon Basin of northeastern Peru. Yurimaguas is a city of ≈63,000 inhabitants located ≈184 meters above sea level and ≈388 km southwest of Iquitos.

### Febrile Surveillance Study Population

The human use study protocols were approved by the Peruvian Ministry of Health and by the NMRC Institutional Review Board (protocol NMRCD.2000.0006). Study subjects enrolled were >5 years of age and sought treatment for an acute, febrile, undifferentiated illness at military or civilian outpatient clinics at predetermined study sites. The criteria for inclusion of patients were fever >38°C of no more than 5 days in duration and nonspecific symptoms, such as headache, fatigue, or myalgia. Demographic and clinical information were obtained from each patient at the time of voluntary enrollment, and patients >18 years of age signed individual consent forms. Paired blood samples were collected, the first during the acute phase of illness and the second 2–4 weeks after symptom onset.

### Virus Isolation and Serologic Assays

Serum samples collected during the acute phase of illness were used to isolate viruses by cell culture techniques, and samples collected during both acute and convalescent phases of illness were assayed by using IgM ELISA for evidence of arboviral infections (*9*). The procedure used for virus isolation was described by Aguilar et al. (*8*). In brief, serum samples were inoculated into flasks containing either confluent monolayers of African green monkey kidney cells of the Vero lineage or *Aedes albopictus* mosquito (C6/36) cells and maintained at 37°C and 28°C, respectively. The cell cultures were examined daily for 10 days for evidence of viral cytopathic effects (CPE); on the appearance of CPE, spot-slides were prepared and an immunofluorescence assay was done by using a polyclonal antibody against specific arboviruses that are known to circulate in Peru (*9*).

Prototype group C viruses were inoculated into flasks containing confluent monolayers of African green monkey Vero kidney cells and maintained at 37°C, as described previously. On the appearance of CPE, cell culture supernatants were harvested and clarified by centrifugation, and viral RNA was extracted.

### Extraction of Viral RNA

For the extraction of RNA, cell culture supernatants were harvested and clarified by low-speed centrifugation (2,000 × *g*, 10 min at 4°C), filtered through a 0.45-μM pore size filter (EMD Millipore, Billerica, MA, USA), and treated with a combination of DNases: 14 U Turbo DNase (Ambion, Austin, TX, USA); 20 U Benzonase (EMD Millipore); and 20 U RNase One (Promega, Madison, WI, USA) for 1 h at 37°C. Next, 24 mL of supernatant was loaded on top of 8 mL 30% sucrose (in TEN, pH 7.4), and centrifuged at 15,000 × *g* for 4 h at 4°C. Finally, the pellet was resuspended in 250 μL RNase/DNase and protease-free water (Ambion), and viral RNA was extracted using the Trizol reagent (Invitrogen, Carlsbad, CA, USA) by using the manufacturer’s protocols.

### Next-Generation Sequencing and Phylogenetic Analyses

Viral RNA (≈0.9 μg) was fragmented by incubation at 94°C for 8 min in 19.5 μL of Illumina fragmentation buffer 15016648 (Illumina, San Diego, CA, USA). A sequencing library was prepared from the sample RNA by using an Illumina TruSeq RNA Sample Preparation Kit v2 using the manufacturer’s protocol (Illumina). The sample was sequenced on a HiSeq 1000 by using the 2 × 50 paired-end protocol. Reads in fastq format were quality filtered, and any adaptor sequences were removed by using Trimmomatic software (*19*). The de novo assembly program ABySS (*20*) was used to assemble the reads into contigs, using several different sets of reads and k values from 20 to 40. In all samples, host reads were filtered out before de novo assembly. The longest contigs were selected, and reads were mapped back to the contigs by using bowtie2 (*21*) and visualized with the Integrated Genomics Viewer (*22*) to verify that the assembled contigs were correct. Total reads ranged from 1.5 to 12 million; the percentage of reads mapping to the virus genome in each sample ranged from 12% to 33%. (Details are available upon request from the authors.) 

We deposited the complete genome sequences of Itaya virus and other group C viruses obtained for this study in GenBank under accession numbers KM092512-KM092514 and KM280924-KM280938. We used the neighbor-joining method available in MEGA5 (*23*) for phylogenetic analysis. The support for each node was determined by using 1,000 bootstrap replicates.

### Antigenic Characterization

We used established methods to obtain hyperimmune ascitic fluid for classic cross-neutralization tests (*24*). Mice received 4 weekly intraperitoneal injections of 10% virus-infected newborn mouse brain suspension with Freud’s adjuvant. Sarcoma 180 cells were also given intraperitoneally with a final injection to induce ascites formation. Antigenic differences among the viruses were then investigated by using cross-neutralization assays (*25*).

## Results

### Identification of IQT9646 as a Novel Reassortant *Orthobunyavirus*

In 1999, the virus strain IQT9646 was isolated from a 25-year-old male febrile patient who resided in Belen, Iquitos, Peru. The patient had an illness with symptoms of fever, headache, retro-orbital pain, arthralgia, chills, cough, and nasal congestion. These clinical symptoms are also characteristic of dengue, malaria, and other tropical infectious diseases common in the region (*26*,*27*). The strain was initially classified as a Maguari isolate based on the results of serologic reactivity in an indirect immunofluorescence test. Maguari virus has been previously isolated from mosquitoes of the *Aedes, Mansonia*, and *Psorophora* spp. in Brazil; a variety of other mosquito species in Ecuador, Brazil, Trinidad, Colombia, Argentina, and French Guiana; and from horses in Guyana and Colombia (*28*,*29*). Nevertheless, evidence of human infection with Maguari virus is lacking. Therefore, we attempted to further identify and genetically characterize this strain using primers specific for different orthobunyaviruses, including Maguari; however, our attempts to amplify partial or complete genomic segments were unsuccessful. We therefore sought to obtain the complete sequences of the S, M, and L segments using an unbiased sequencing approach, then phylogenetic analyses to determine the relationship of the IQT9646 isolate to other viruses within the *Orthobunyavirus* genus. 

**Figure 2:**
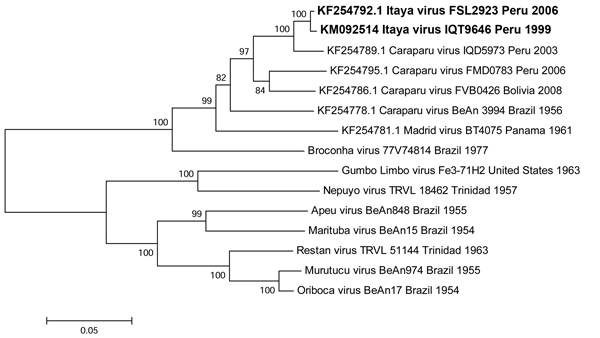
Neighbor-joining phylogenetic tree of group C orthobunyaviruses constructed by using MEGA5 (*23*) on the basis of the small gene segments of published virus sequences and Itaya virus strains isolated in Peru in 1999 and 2006 (boldface). The Itaya strain segments show a close relationship to Caraparu virus. Virus strains are labeled by code designation. Numbers indicate bootstrap values for the clades to the right. Bootstrap values were obtained based on 1,000 replicates. Scale bar indicates nucleotide substitutions per site.

**Figure 3:**
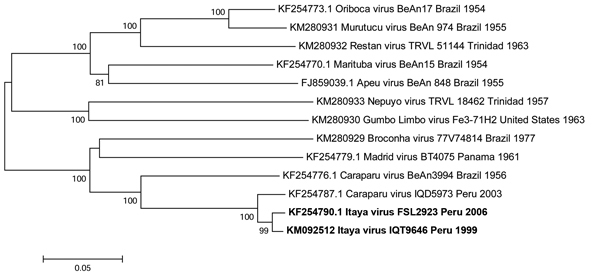
Neighbor-joining phylogenetic tree of group C orthobunyaviruses constructed by using MEGA5 (*23*) on the basis of the large gene segments of published virus sequences and Itaya virus strains isolated in Peru in 1999 and 2006 (boldface). The Itaya strain segments show a close relationship to Caraparu virus. Virus strains are labeled by code designation. Numbers indicate bootstrap values for the clades to the right. Bootstrap values were obtained based on 1,000 replicates. Scale bar indicates nucleotide substitutions per site.

**Figure 4:**
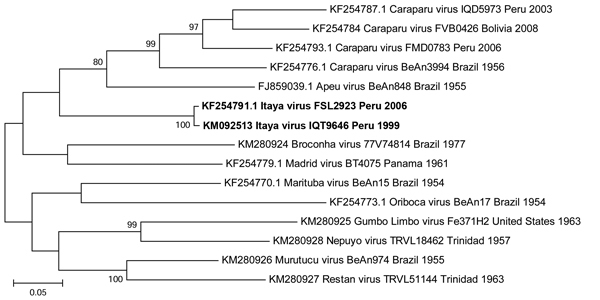
Neighbor-joining phylogenetic tree of group C orthobunyaviruses constructed by using MEGA5 (*23*) on the basis of the large gene segments of published virus sequences and Itaya virus strains isolated in Peru in 1999 and 2006 (boldface). The Itaya strain segments show a more distant relationship to Caraparu virus than for the small and large segments, indicating that Itaya virus is a novel reassortant strain. Virus strains are labeled by code designation. Numbers indicate bootstrap values for the clades to the right. Bootstrap values were obtained based on 1,000 replicates. Scale bar indicates nucleotide substitutions per site.

Phylogenetic trees based on the S and L gene segments placed the IQT9646 virus among isolates of Caraparu virus, a member of the group C virus serogroup (Figures 2, 3), a pathogen known to cause febrile illness in humans and animals in the Amazon region of Peru (*9*,*11*). However, the M segment phylogenetic tree indicated that the IQT9646 virus had an M segment sequence divergent from Caraparu. In an attempt to determine the source of the M segment, we used a comprehensive full-genome sequence approach of other group C viruses and determined that the M segment of the IQT9646 strain was not closely related to other group C viruses but instead was most similar to the Caraparu and Apeu viruses (Figure 4). The S and L segment fragment sequences of the IQT9646 strain exhibited 97.6% and 96.6% nucleotide identity, respectively, to the prototype Caraparu strain BeAn3994 and to the Peruvian Caraparu strain IQD5973, whereas the M segment sequence displayed ≈75% nucleotide identity to the prototype and Peruvian Caraparu strains. In summary, these results suggest that IQT9646 is possibly a group C reassortant virus.

### Antigenic Characterization of IQT9646

To determine if the IQT9646 strain is antigenically distinct from Caraparu virus, we investigated the serologic relationships of IQT9646 using cross-neutralization tests. Mouse antisera were prepared against the IQT9646 strain and the prototype Caraparu strain BeAn 3994. These antisera displayed a minimum 4-fold difference in neutralization titers between these viruses, indicating that IQT9646 is serologically distinct from the prototype Caraparu virus. Because some genetic variation exists between the prototype Caraparu and recent Caraparu isolates from Peru (*11*) that may translate into minor antigenic differences, we included in our analyses a recent Caraparu strain isolated in Peru. The results were consistent with those obtained with the prototype Caraparu strain (Table 2). In summary, we found that the IQT9646 strain is antigenically distinct from Caraparu virus.

**Table 2:** Neutralization titers for IQT9646 and Caraparu viruses by using mouse antisera to determine antigenic differences, Peru*

Strain (virus)	Anti-IQT9646	Anti-BeAn3994
IQT9646 (Itaya)	2,560	80
FPI 2066 (Caraparu)	<40	2,560
BeAn3994 (Caraparu)	40	1,280

*Neutralization titers are expressed as the dilution of mouse immune ascitic fluid antiserum inhibiting 80% of plaque forming units.

### IQT9646 Virus in the Amazon Region of Peru

Genomic characterization of some group C prototype viruses and other isolates found in South America were recently reported (*11*). The strain FSL2923, isolated from a febrile patient residing in Yurimaguas, Peru, in 2006 was found to possess L and S RNA segments closely related to Caraparu virus; the M segment was only ≈75% identical to the M segment of Caraparu virus (*11*). However, this strain was not characterized antigenically, causing uncertainty of whether this virus strain was distinct from Caraparu virus. 

Considering our findings with the IQT9646 strain, which, like FSL2923, possesses the S and L RNA segment sequences closely related to Caraparu virus and the M RNA segment sequence derived from an unidentified group C virus, we made genetic comparison and observed that the S, M, and L RNA segments of IQT9646 and FSL2923 shared >98% nucleotide and >99% amino acid sequence homology for all 3 viral RNA segments. These findings indicate that the FSL2923 isolate is the same virus as strain of IQT9646. The data also suggest that this reassortant virus is widely distributed throughout the Amazon basin in northeastern Peru, because it was isolated in Iquitos and Yurimaguas, in the Department of Loreto, within a 7-year time period (Figure 1).

## Discussion

Arboviral diseases continue to be a frequent cause of illness and death worldwide. In recent years, a considerable number of novel arboviruses associated with outbreaks of human or livestock disease have been identified, and the expansion of known arboviruses into new geographic areas have also been reported (*8*,*30*–*33*). Of great concern is the potential that arboviruses will spread into novel geographic regions with completely naive human and animal populations, changing their patterns of illness as they move across the globe. Another major concern is the possibility that relatively benign viruses with segmented genomes could reassort, resulting in increased pathogenicity. Therefore, because of the public health impact that arboviral diseases continue to have around the globe, there is an urgent need to reinforce surveillance systems to identify the emergence of novel arboviruses and to monitor the activity of existing viruses and their potential expansion across geographic areas.

Surveillance studies performed by NAMRU-6 over the past 2 decades have been instrumental in characterizing the extent of many endemic tropical diseases throughout Latin America (*8*,*34*–*37*). Among the bunyaviruses, Oropouche, Iquitos, Guaroa, and members of the group C viruses were found to account for ≈2.5% of all febrile cases (*8*,*9*). Although the percentage of cases appears relatively low, it is likely that most of human cases are undoubtedly going unrecognized or misdiagnosed as dengue, malaria, or other common acute tropical infectious diseases. This scenario is likely related to laboratory testing limitations and lack of extensive surveillance networks.

Among the bunyaviruses, the average symptomatic rates in Iquitos for group C and Iquitos viruses were 14.3/100,000 and 14.2/100,000, respectively, based on participants enrolled in NAMRU-6’s febrile surveillance protocol (*8*,*9*). Because group C viruses include several human pathogens, recent studies have attempted to genetically characterize a few group C viruses isolated from humans. These efforts identified Caraparu and Marituba viruses and associated them with febrile human disease in Peru (*9*,*11*). However, >10 of the 13 distinct group C viruses have been associated with human disease elsewhere, including Oriboca, Itaqui, Nepuyo, Apeu, Murutucu, Restan, Ossa, Madrid, Caraparu, and Marituba viruses (*38*).

In this study, we aimed to expand our knowledge and understanding of bunyaviruses associated with febrile human illness in Peru, and these efforts focused on Maguari-like viruses, which were provisionally identified by using indirect immunofluorescence assays. Although indirect immunofluorescence assay is a procedure commonly used in laboratories to tentatively identify viruses, there is a degree of cross-reactivity among viruses from the same family; therefore, additional testing is needed to properly identify the viral agent. Therefore, we used a genetic and antigenic approach to characterize and identify the Maguari-like virus isolated from samples of febrile patients. These efforts yielded the identification of the group C reassortant virus that we named Itaya virus. Furthermore, we generated complete genome sequence information for prototype group C viruses, and as noted (*13*), we also observed discrepancies with the Nepuyo, Restan, Murutucu, and Gumbo Limbo virus sequences when compared to those described by Nunes et al. (*39*). We still do not know the reasons for these discrepancies; however, Nunes et al. have acknowledged errors in their sequences and their plans to revise them (*40*), which should eventually clarify these discrepancies.

The prototype strain of Itaya virus was isolated from a patient who resided in Iquitos. The patient visited the health post in January 1999 with a mild febrile illness characterized by fever, headache, retro-orbital pain, arthralgia, and chills, among other symptoms. During February 1999, a patient with similar signs and symptoms visited the same health post in Iquitos, and subsequent studies determined that the patient had been infected with a novel reassortant orthobunyavirus, which we subsequently named Iquitos virus (*8*). The emergence of 2 novel reassortant orthobunyaviruses raises some questions as to what ecologic and environmental conditions favored the reassortment events leading to an emergence of novel human pathogens and their recognition in close succession. Because our limited serologic data suggests that the current arbovirus diagnostic tests fail to detect IgM antibodies produced in response to Itaya virus (data not shown), retrospective studies of febrile cases using Itaya virus specific tests may help determine the public health effects this pathogen may have in the area before and after its first isolation. The fact that another strain of Itaya virus was isolated in 2006 from another region within the northeastern Amazon Basin suggests that the virus may have caused more febrile human cases than previously recognized. Therefore, Itaya virus should be included in the list of potential pathogens that may account for a percentage of the 67% febrile cases enrolled in ongoing passive surveillance that are currently undiagnosed (*9*). Additional epidemiologic and ecologic studies are also needed to determine how widespread the virus is within the Amazon region and in neighboring areas and to identify potential vectors and reservoirs involved in the transmission of Itaya and other group C viruses. 

In conclusion, our report expands upon the list of known arboviruses associated with febrile illness in Peru and raises awareness about the continuous emergence of reassortant bunyaviruses with human pathogenic potential. Future studies designed to genetically characterize existing and recently isolated bunyaviruses may be able to identify the viral donor of the Itaya M segment.
